# Long-term risk of mortality for acute kidney injury in HIV-infected patients: a cohort analysis

**DOI:** 10.1186/1471-2369-14-32

**Published:** 2013-02-11

**Authors:** José António Lopes, Maria João Melo, Mário Raimundo, André Fragoso, Francisco Antunes

**Affiliations:** 1 Department of Nephrology and Renal Transplantation, Hospital de Santa Maria, Centro Hospitalar Lisboa Norte, EPE, Av. Prof. Egas Moniz, Lisboa, Portugal; 2 Department of Nephrology, Hospital de Faro, Faro, Portugal; 3 Department of Infectious Diseases, Hospital de Santa Maria, Centro Hospitalar Lisboa Norte, EPE, Av. Prof. Egas Moniz, Lisboa, Portugal

**Keywords:** Acute kidney injury, HIV, Mortality

## Abstract

**Background:**

Acute kidney injury (AKI) is common in hospitalized human immunodeficiency virus (HIV)-infected patients and is associated with hospital mortality. We aimed to evaluate the impact of AKI on long-term mortality of hospitalized HIV-infected patients.

**Methods:**

Retrospective analysis of a cohort of 433 hospitalized HIV-infected patients who were discharged alive from the hospital. AKI was defined according to ‘Risk Injury Failure Loss of kidney function End-stage kidney disease’ creatinine criteria, as an increase of baseline serum creatinine (SCr) X 1.5 or in patients with baseline SCr > 4 mg/dL if there was an acute rise in SCr of at least 0.5 mg/dL. Cumulative mortality curves were determined by the Kaplan-Meier method, and log-rank test was employed to analyze statistically significant differences between curves. Cox regression method was used to determine independent predictors of mortality. Risk factors were assessed with univariate analysis, and variables that were statistically significant (P < 0.05) in the univariate analysis were included in the multivariate analysis.

**Results:**

Sixty-four patients (14.8%) had AKI. Median follow-up was 37 months. At follow-up 81 patients (18.7%) died. At 1, 2 and 5 years of follow-up, the cumulative probability of death of patients with AKI was 21.2, 25 and 31.3%, respectively, as compared with 10, 13.3 and 16.5% in patients without AKI (log-rank, P = 0.011). In multivariate analysis AKI was associated with increased mortality (adjusted HR 1.7, 95% CI 1.1-3; P = 0.049).

**Conclusions:**

AKI was independently associated with long-term mortality of hospitalized HIV-infected patients.

## Background

Although acute kidney injury (AKI) is a common complication in the hospital and has an immediate impact on morbidity, mortality, and resource utilization [1-4], its detrimental effect appears to persist also after recovery, since AKI has been associated with an increased long-term mortality risk [5-7]. Understanding the impact of AKI on long-term outcomes will have a marked impact on treatment and risk stratification during hospitalization and will assist with guiding follow-up care after discharge.

Over the last two decades, the number of individuals infected with human immunodeficiency virus (HIV) has markedly increased, and, actually, > 30 million people are affected with HIV infection worldwide [8]. Since the introduction of the highly active antiretroviral therapy (HAART) at the end of 1995, the annual number of deaths reported with HIV infection decreased dramatically as well as the number of deaths caused by HIV infection or by an acquired immunodeficiency syndrome (AIDS)-defining disease. Conversely, comorbidities such as kidney disease, liver disease, heart disease and non-AIDS-defining cancers have proportionally increased and have become significant contributors to morbidity in HIV-infected patients [9-11].

Renal disorders in HIV-infected patients can present as an acute or chronic condition, and they are associated with increased morbidity and mortality in this population [12-16]. Acute kidney injury (AKI) is a common complication in ambulatory HIV-infected patients treated with HAART and has been associated with prior renal impairment, lower CD4 levels, AIDS, hepatitis C virus coinfection and liver disease [17,18]. HIV-infected patients are also at increased risk for AKI development within hospitalization, related to volume depletion, sepsis and the acute administration of nephrotoxic medications or radiocontrast. Before the advent of HAART, studies addressing AKI on HIV-infected patients typically included only severe cases of AKI which were identified through hospital records or biopsy databases [19-21]. In the HAART era, the reported incidence of AKI in hospitalized HIV-infected patients ranged from 6 to 20%, and AKI was associated with increased in-hospital mortality [22,23]. In the present study, we evaluated the impact of AKI on long-term mortality in a cohort of hospitalized HIV-infected patients who were discharged alive from the hospital.

## Methods

The present study was retrospective, including HIV-infected patients admitted to the Department of Infectious Diseases of the Hospital de Santa Maria (Lisbon, Portugal) between January 2005 and December 2007. Hospital de Santa Maria is a tertiary and teaching hospital providing medical assistance to an area with almost 3000000 inhabitants. Since this was a retrospective and observational study that did not evaluate a specific therapeutic or prophylactic intervention, study approval was waived by the Ethical Committee of our Hospital according to Institutional guidelines.

### Selection criteria, population and study periods

During the study period, 547 HIV-infected patients were admitted to the Department of Infectious Diseases of our hospital; 13 of them were chronic kidney disease (CKD) patients on dialysis and were not included in this study. None of the patients had received a renal transplant. From the 534 remaining patients, 45 patients who were hospitalized <24 h and/or had no serum creatinine (SCr) determination within hospitalization and 56 patients who died within the hospitalization were excluded from the analysis. Therefore, in this study, we focused on 433 hospitalized HIV-infected who were alive at hospital discharge (mean age: 41.5 years; 290 Male; 312 Caucasian; 412 infected with HIV-1, 18 with HIV-2 and 3 with both HIV-1 and HIV-2) and analyzed them as a cohort. In 285 patients (65.8%) HIV was diagnosed previously to the hospitalization and in 148 patients (34.2%) HIV was only diagnosed within the hospitalization (86% of them were late presenters). From those patients with previously documented HIV infection, the majority (N = 250, 87.7%) were followed-up by a specialist (70% were receiving HAART) while 12.3% of patients were not followed-up by a specialist.

### Definitions

Acute kidney injury (AKI) was defined according to ‘Risk Injury Failure Loss of kidney function End-stage kidney disease’ creatinine criteria [24], as an increase of baseline serum creatinine (SCr) X 1.5. AKI was also considered in patients with baseline SCr > 4 mg/dL in whom there was an acute rise in SCr of at least 0.5 mg/dL (irrespective of the percentual increase in SCr) as these patients are also classified as having AKI (Failure class).The most recent SCr value registered within 1–6 months prior to admission was considered the baseline SCr and was available in 417 patients (96.3%); when it was unavailable (N = 16, 3.7%) baseline SCr was estimated by the Modification of Diet in Renal Disease equation [25], as recommended (assuming a lower limit of the normal baseline glomerular filtration rate (GFR) of 75 mL/min/1.73m^2^) and previously applied [1,4,26]. Complete renal function recovery was considered if the SCr at hospital discharge with reference to baseline SCr was lower than X 1.5, and in patients with baseline SCr >4 mg/dL, complete renal function recovery was also considered if SCr at hospital discharge was also <0.5 mg/dL with reference to baseline SCr. On the other hand, partial renal function recovery was considered if the SCr at hospital discharge with reference to baseline SCr was equal or higher than X 1.5, and in patients with baseline SCr >4 mg/dL, partial renal function recovery was also considered if SCr at hospital discharge was also ≥0.5 mg/dL with reference to baseline SCr; in both cases, there was no dialysis requirement at hospital discharge [15]. HIV infection was staged according to the Centers for Disease Control and Prevention (CDC) classification [27]. Diabetes mellitus was diagnosed according to the World Health Organization criteria [28], and hypertension was diagnosed based on the seventh report of the Joint National Committee (JNC 7) [29]. Cardiovascular disease (CVD) included chronic heart failure, coronary artery disease, cerebrovascular disease and peripheral arterial disease. Chronic kidney disease (CKD) was considered whenever baseline GFR was < 60 mL/min/1.73m^2^[30]. Chronic lung disease included emphysema, chronic bronchitis and asthma. Patients with cancer included those patients with malignant solid or hematopoietic neoplasm. Sepsis was diagnosed in accordance with the American College of Chest Physicians and the Society of Critical Care Medicine consensus [31].

### Variables

Manual patient medical charts and electronic hospital database were reviewed by three investigators (MJM, MR and AF) to study demographic data (age, gender, race), comorbidity (diabetes mellitus, hypertension, CVD, CKD, chronic lung disease, cirrhosis, hepatitis C virus and hepatitis B virus coinfections and cancer), HIV-related characteristics (type of HIV, antiretroviral therapy, CD4 cell count, HIV viral load, stage of HIV infection), SCr, sepsis occurrence, intensive care unit (ICU) admission, etiology of AKI, need for renal replacement therapy (RRT), length of hospital stay and mortality.

The investigators determined the etiology of AKI based on the temporal relationship between the occurrence of AKI and its causes. When more than one attributable cause of AKI was identified the etiology of AKI was considered multifactorial.

### Statistical analysis

Continuous variables are expressed as mean (standard deviation) and categorical variables as percentage of number of cases. Comparisons between patients with and without AKI were performed using the Student’s t-test and the χ2 test, respectively, for continuous and categorical variables. Multivariate logistic regression analysis was used to determine risk factors of AKI; only statistically significant variables in the univariate analysis were entered in the multivariate analysis.

Only the first hospitalization was analyzed for patients with multiple admissions. Vital status was ascertained for all patients through hospital database (in 85% of the cases) and phone call to patients or their family by using contact details provided on hospital database (in 15% of cases). Cumulative mortality curves were determined by the Kaplan-Meier method, and log-rank test was employed to analyze statistically significant differences between curves. Cox regression method was used to determine independent predictors of mortality. Risk factors were assessed with univariate analysis, and variables that were statistically significant (P < 0.05) in the univariate analysis were included in the multivariate analysis by applying a multiple forward stepwise Cox regression. Data are presented as hazard ratios (HRs) with 95% confidence intervals (CIs). A two-tailed P-value < 0.05 was considered significant. Analysis was performed with the statistical software package SPSS 19.0 for Windows (Produtos e Serviços de Estatísticas, Lisboa, Portugal).

## Results

Sixty-four patients (14.8%) had AKI during hospital stay. Median time to the occurrence of AKI was 8 days (1–59 days). Patients who developed AKI were more likely to have preexisting hypertension (P = 0.034), preexisting CKD (P = 0.001) and AIDS (P = 0.003) and nephrotoxic drugs administration (P < 0.0001) and radiocontrast use (P = 0.017) were more often in those patients. Furthermore, sepsis was more prevalent in patients with AKI (P < 0.0001) and these patients had lengthened time of hospitalization (P < 0.0001), as compared with patients with no acute renal impairment. Individuals with AKI were more likely to have required ICU admission (P = 0.006) (Table 1). AIDS (adjusted OR 2.6, 95% CI 1.1-5.9, P = 0.029), nephrotoxic drugs (adjusted OR 4.6, 95% CI 2.1-10.3, P < 0.0001) and sepsis (adjusted OR 28.8, 95% CI 12.2-67.8, P < 0.0001) were independently associated with increased risk for AKI.

**Table 1:** Baseline characteristics of 433 hospitalized HIV-infected patients who were alive at hospital discharge

**Variable**	**No AKI**	**AKI**	**P-value**
	**(N=369)**	**(N=64)**	
Age (years)	41.3 (11.7)	42 (10.4)	0.647
Gender (Male)	248 (67.2%)	42 (65.6%)	0.804
Race (Caucasian)	267 (72.3%)	45 (70.3%)	0.736
Comorbidity			
Diabetes mellitus	21 (5.7%)	3 (4.7%)	0.746
Hypertension	36 (9.8%)	12 (18.8%)	0.034
CVD	19 (5.1%)	3 (4.7%)	0.877
CKD	7 (1.9%)	6 (9.4%)	0.001
Chronic lung disease	11 (2.9%)	0 (0%)	0.162
Cirrhosis	32 (8.7%)	9 (14.1%)	0.174
HCV coinfection	124 (33.6%)	20 (31.3%)	0.712
HBV coinfection	25 (6.8%)	8 (12.5%)	0.111
Cancer	30 (8.1%)	6 (9.4%)	0.739
HIV-related characteristics			
Type of HIV (HIV-1)	349 (94.6%)	63 (98.4%)	0.185
HAART	149 (40.4%)	26 (41.6%)	0.971
CD4 count < 200 cells/mm^3^	215 (58.3%)	43 (67.2%)	0.179
Viral load detectable	313 (84.8%)	56 (87.5%)	0.578
AIDS	236 (63.9%)	58 (82.8%)	0.003
Renal function			
Baseline SCr	0.9 (0.3)	0.9 (0.5)	0.929
SCr at hospital discharge	0.8 (0.3)	1.4 (0.9)	<0.0001
Sepsis	12 (3.3%)	32 (50%)	<0.0001
ICU admission	12 (3.3%)	7 (10.9%)	0.006
Nephrotoxic drugs	35 (9.5%)	23 (35.9%)	<0.0001
Radiocontrast use	37 (10.3%)	13 (20.3%)	0.017
Length of hospital stay (days)	20 (20)	35 (24)	<0.0001

AIDS- acquired immunodeficiency syndrome. CKD- chronic kidney disease, considered whenever baseline glomerular filtration rate was <60 mL/min/1.73m^2^. CVD- cardiovascular disease. HAART- highly active antiretroviral therapy. HBV- hepatitis B virus. HCV- hepatitis C virus. HIV- human immunodeficiency virus. ICU- intensive care unit. SCr- serum creatinine.

In all cases, there was at least an obvious cause for the development of AKI which was multifactorial in 22 patients (34.4%). The most common etiologies of AKI were sepsis (N = 32; 50%), nephrotoxic drugs administration (N = 23, 35.9%), volume depletion (N = 16, 25%), use of radiocontrast (N = 13, 20.3%) and rhabdomyolysis (N = 6, 9.4%). Other less common causes of AKI were acute urinary tract obstruction (N = 1) and thrombotic microangiopathy (N = 1). Of interest, only one patient was submitted to renal biopsy which revealed thrombotic microangiopathy.

Six of the 64 patients with AKI (9.4%) underwent RRT; all of them received intermittent hemodialysis. The mean duration of dialysis treatments was 10 (9) days.

**Figure 1:**
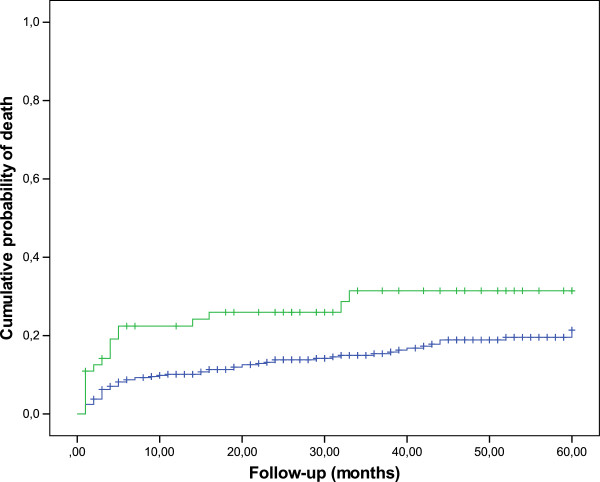
**Cumulative probability of death. **Cumulative probability of death in patients with (green line) and without (blue line) acute kidney injury during previous hospitalization; log-rank test, P = 0.011.

Median follow-up was 37 months. At follow-up 81 patients (18.7%) died. The probability of death significantly differed among patients with AKI and without AKI during previous hospitalization (Figure 1). At 1, 2 and 5 years of follow-up, the cumulative probability of death of patients with AKI was 21.2, 25 and 31.3%, respectively, as compared with 10, 13.3 and 16.5% in patients without AKI (log-rank, P = 0.011) (Figure 1). In multivariate analysis AKI was associated with increased mortality (adjusted HR 1.7, 95% CI 1.1-3; P = 0.049) (Table 2). The causes of death among AKI patients (N = 18) were sepsis in thirteen patients (72.2%), neoplasm in one patient (5.6%), gastrointestinal bleeding in one patient (5.6%), and it was unknown in three patients (16.7%); sepsis (N = 30; 47.6%), neoplasm (N = 13; 20.6%), cerebrovascular disease (N = 2; 3.2%), progressive multifocal leukoencephalopathy (N = 2; 3.2%), liver failure (N = 1; 1.6%), gastrointestinal bleeding (N = 1; 1.6%), caquexia (N = 1; 1.6%), and undetermined (N = 12; 19%) were the causes of death among patients without AKI (N = 63).

**Table 2:** Univariate and multivariate analysis including acute kidney injury to determine predictors of mortality

**Variable**	**Unadjusted**	**P-value**	**Adjusted**	**P-value**
	**HR (95% CI)**		**HR (95% CI)**	
Age (years)	1.01 (0.9-1.03)	0.110		
Gender (Male)	1.3 (0.8-2.1)	0.322		
Race (Caucasian)	0.9 (0.6-1.6)	0.962		
Comorbidity				
Diabetes mellitus	1.4 (0.6-3.3)	0.413		
Hypertension	1.1 (0.5-2.1)	0.848		
CVD	1.2 (0.5-3.0)	0.651		
CKD	2.7 (1.1-6.6)	0.034	2 (0.8-5.2)	0.147
Chronic lung disease	0.9 (0.2-4.0)	0.968		
Cirrhosis	0.6 (0.2-1.5)	0.295		
HCV coinfection	1.5 (0.9-2.3)	0.074		
HBV coinfection	1.5 (0.7-3.1)	0.273		
Cancer	2.5 (1.3-4.6)	0.004	2.1 (1.1-4)	0.017
HIV-related characteristics				
Type of HIV (HIV-1)	2.1 (0.5-8.4)	0.312		
HAART	1.1 (0.6-1.6)	0.963		
CD4 count < 200 cells/mm^3^	1.6 (1.1-2.6)	0.039	1.4 (0.8-2.4)	0.197
Viral load detectable	1.6 (0.8-3.0)	0.196		
AIDS	1.8 (1.1-3.0)	0.023	1.3 (0.8-2.4)	0.311
Sepsis	1.6 (0.8-2.9)	0.176		
ICU admission	0.5 (0.1-2)	0.317		
AKI	1.9 (1.1-3.3)	0.013	1.7 (1.1-3)	0.049

AIDS- acquired immunodeficiency syndrome. AKI- acute kidney injury. CI- confidence interval. CKD- chronic kidney disease, considered whenever baseline glomerular filtration rate was <60 mL/min/1.73m^2^. CVD- cardiovascular disease. HAART- highly active antiretroviral therapy. HBV- hepatitis B virus. HCV- hepatitis C virus. HIV- human immunodeficiency virus. HR- hazard ratio. ICU- intensive care unit.

Forty-three patients (67.2%) with AKI had complete renal function recovery, 19 patients (29.7%) with AKI had partial renal function recovery and two patients (3.1%) with AKI were dialysis-dependent at hospital discharge. Mortality did not differ between AKI patients with complete renal function recovery and AKI patients who did not have complete renal function recovery at hospital discharge (27.9% versus 28.6%, P = 0.956).

## Discussion

We conducted a retrospective study of a cohort of 433 hospital surviving HIV-infected patients to determine the impact of AKI during hospitalization on long-term mortality. We found that AKI had a negative impact on long-term mortality (median follow-up, 37 months) of these patients. As compared with patients without AKI, patients with AKI had a 1.7-fold risk of death. These findings expand on results from previous studies that showed the increased risk of short-term death associated with AKI in hospitalized HIV-infected patients [22,23].

It has been shown that patients who survive AKI have a greater rate of long-term mortality and other adverse outcomes than patients who survive hospitalization without AKI, in varied settings, such as community/hospitalized patients, hospitalized patients, ICU patients, septic patients, cardiac surgery, patients undergoing aortic stent placement or percutaneous coronary intervention, liver transplant, lung transplant and hematopoietic cell transplant [32-34].

To our knowledge, this is the second study that reports long-term mortality of AKI in hospitalized HIV-infected patients. Recently, Choi and colleagues evaluated the long-term consequences of AKI in HIV-infected persons. For this purpose, they studied 17.325 patients in a national HIV registry during their first hospitalization. Over a mean follow-up period of 5.7 years, they found that AKI was associated with increased mortality and there was a graded relationship between severity of AKI and mortality. Furthermore, long-term risk for heart failure, cardiovascular disease and end-stage renal disease was also higher in patients who developed AKI during hospitalization [35].

The mechanism by which AKI contributes to increased long-term mortality is not completely understood. After an episode of AKI, it is likely that there is failure to resolve renal structure and function adequately [36,37]. Acute kidney injury itself may increase the risk of subsequent events and decrease kidney reserve leading to an increased risk of CKD. AKI also leads to coagulation abnormalities and increased incidence of sepsis with multi-organ failure as well as to upregulation of cytokines (interleukin-1, interleukin-6, tumor necrosis factor-α) and/or immune-mediated major organ dysfunction (i.e. heart, lungs, and brain) [38,39]. It should be remembered that in the present study sepsis was a more prevalent cause of death in patients with AKI compared with those patients without AKI (72.2% versus 47.6%, respectively). Similar mechanisms have been directly associated with HIV infection. In fact, HIV leads to chronic immune activation and a prothrombotic state, which also features increases in interleukin-1, interleukin-6, and tumor necrosis factor-α which have been implicated in the pathogenesis of atherosclerosis, progression to AIDS, and mortality in HIV-infected persons [40-42]. We hypothesize that AKI may magnify these derangements through these common mechanisms.

In the present study, mortality did not differ among AKI patients with and without complete renal function recovery. These results should be interpreted with extreme caution, since the number of patients may be insufficient to detect a difference on outcome. However, the similar outcome between AKI patients who did or not recover renal function could also be explained by the permanent injury to other vital organs caused by AKI, thus affecting future survival, despite the reversible nature of clinical AKI in which creatinine level returns to baseline after the episode [38,39].

Our study has some limitations. First, the single-center nature of the study largely limits its generalizability, and its retrospective design with a relatively small cohort of patients did not allow the evaluation of other confounders with prognostic importance. Second, we did not have daily values of SCr and urine output records; therefore, we were not able to capture all cases of AKI which occurred within the hospitalization. Third, we were not able to determine patients` adherence to HAART. Altogether, the high number of patients with no previous diagnosis of HIV infection or without previous followed-up by a specialist and the eventual non-adherence to HAART could explain at least in part low CD4 count and high viral load. Fourth we did not analyze the long-term impact of AKI on cardiovascular disease and end-stage renal disease.

Despite the aforementioned constraints, however, our study has some virtues. First, AKI was diagnosed by the RIFLE criteria based on SCr determinations. Second, contrary to other studies [35], the causes of AKI and the causes of death were determined.

## Conclusion

AKI was independently associated with long-term mortality of hospitalized HIV-infected patients and, therefore, these patients should be carefully monitored following hospital discharge.

## Abbreviations

AIDS: Acquired immunodeficiency disease syndrome; AKI: Acute kidney injury; CI: Confidence interval; CKD: Chronic kidney disease; CVD: Cardiovascular disease; GFR: Glomerular filtration rate; HAART: Highly active antiretroviral therapy; HIV: Human immunodeficiency virus; HR: Hazard ratio; ICU: Intensive care unit; RIFLE: Risk Injury Failure Loss of kidney function End-stage kidney disease; SCr: Serum creatinine.

## Competing interests

The authors declare that they have no competing interests.

## Authors’ contributions

JAL have made substantial contributions to conception and design, analysis and interpretation of data, as well as has been involved in drafting the manuscript and revising it critically for important intellectual content. MJM and MR have collected data. FA has revised the manuscript critically for important intellectual content. All authors have given final approval of the version to be published. All authors read and approved the final manuscript.

## Pre-publication history

The pre-publication history for this paper can be accessed here:

http://www.biomedcentral.com/1471-2369/14/32/prepub
